# Supplementary material for: MetaRibo-Seq measures translation in microbiomes
Source: Nat Commun. 2020 Jun 29;11:3268. doi: 10.1038/s41467-020-17081-z (PMC7324362; doi:10.1038/s41467-020-17081-z)
Supplement: Supplementary file 10 — Supplementary Data 7 [file 41467_2020_17081_MOESM10_ESM.zip › File2/Confidence_High_Taxonomy/82682_out.krona.html]

Javascript must be enabled to view this page.

members
magnitude
magnitudeUnassigned
count
unassigned
taxon
rank

82682\_out

59

59
2
superkingdom

1
phylum
976

1
200643
class

1
171549
order

1
family
815

816
genus
1


SRS019286\_contig\_number\_310
2292923
species
1

58
phylum
1239

species

SRS017307\_contig\_number\_contig-100\_1094.92539
1263007
1

57
186801
class

order
186802
57

57
541000
family

57
genus
216851

57

SRS011084\_contig\_number\_contig-100\_1434.205325SRS011134\_contig\_number\_45674SRS013098\_contig\_number\_46974SRS015264\_contig\_number\_contig-100\_4240.126965SRS015579\_contig\_number\_21595SRS015960\_contig\_number\_contig-100\_22432.62697SRS017859\_contig\_number\_contig-100\_3861.3862SRS018623\_contig\_number\_contig-100\_1165.110163SRS021484\_contig\_number\_32317SRS022609\_contig\_number\_51261SRS024331\_contig\_number\_contig-100\_28274.109044SRS045004\_contig\_number\_contig-100\_24911.53590SRS045645\_contig\_number\_contig-100\_1187.179804SRS046502\_contig\_number\_contig-100\_38515.38516SRS049896\_contig\_number\_contig-100\_6238.116106SRS049959\_contig\_number\_contig-100\_38854.127371SRS050422\_contig\_number\_contig-100\_4610.186123SRS050998\_contig\_number\_17046SRS053573\_contig\_number\_contig-100\_10673.10673SRS056259\_contig\_number\_contig-100\_16011.115737SRS056273\_contig\_number\_contig-100\_2747.201751SRS063190\_contig\_number\_3010SRS074964\_contig\_number\_2135SRS075773\_contig\_number\_31159SRS076756\_contig\_number\_contig-100\_505.134529SRS076929\_contig\_number\_contig-100\_482.152485SRS077194\_contig\_number\_18911SRS097920\_contig\_number\_contig-100\_759.193539SRS097958\_contig\_number\_8920SRS098571\_contig\_number\_contig-100\_2971.371501SRS098881\_contig\_number\_contig-100\_45072.45073SRS101376\_contig\_number\_contig-100\_766.145536SRS101433\_contig\_number\_14520SRS1041037\_contig\_number\_9329SRS1041092\_contig\_number\_contig-100\_10888.10889SRS1041141\_contig\_number\_contig-100\_775.776SRS104636\_contig\_number\_17227SRS104693\_contig\_number\_contig-100\_20252.68023SRS105082\_contig\_number\_14372SRS105153\_contig\_number\_contig-100\_1497.226922SRS140492\_contig\_number\_15784SRS140513\_contig\_number\_8714SRS142980\_contig\_number\_6020SRS143148\_contig\_number\_26442SRS143417\_contig\_number\_contig-100\_3978.175099SRS143876\_contig\_number\_contig-100\_3181.211797SRS144297\_contig\_number\_contig-100\_33405.33406SRS144506\_contig\_number\_6497SRS144537\_contig\_number\_47622SRS144714\_contig\_number\_27166SRS146764\_contig\_number\_30884SRS147139\_contig\_number\_42060SRS147346\_contig\_number\_contig-100\_6906.323821SRS147977\_contig\_number\_10594SRS148784\_contig\_number\_23591SRS149784\_contig\_number\_24268SRS893382\_contig\_number\_contig-100\_4874.4875
853
species
